# Leiomyoma of the seminal vesicle

**DOI:** 10.1590/0100-3984.2016.0159

**Published:** 2018

**Authors:** Tatiane Souza Oliveira, Dimitrius Nikolaos Jaconi Stamoulis, Luis Ronan Marquez Ferreira de Souza, Antonio Carlos Oliveira Meneses, Monise Marques Mori

**Affiliations:** 1 Universidade Federal do Triângulo Mineiro (UFTM), Uberaba, MG, Brazil.; 2 Hospital das Clínicas da Universidade Federal do Triângulo Mineiro (UFTM), Uberaba, MG, Brazil.

*Dear Editor,*

A 60-year-old male patient was admitted to the urology outpatient clinic with an expansile lesion detected by rectal examination. He reported having undergone transurethral resection of the prostate. He described himself as a smoker and an alcoholic. He reported no symptoms of wasting or urinary tract symptoms. Transabdominal pelvic ultrasound showed a heterogeneous prostate with normal contours and of normal weight (Figure 1A). Seminal vesicles were enlarged, resulting in a well-defined, heterogeneous solid lesion. Magnetic resonance imaging (MRI) demonstrated a well-defined, solid-cystic expansile lesion in the right seminal vesicle, with an intermediate signal in a T1-weighted sequence, a hypointense signal in a T2-weighted sequence, and a fluid-fluid level in the sagittal plane. Showing no post-contrast enhancement or restricted diffusion, the lesion extended to the contralateral seminal vesicle and maintained cleavage with the adjacent structures (Figures 1B and 1C). These findings raised the possibility of a benign nodule, maintaining the rationale used for ovarian and renal lesions^(^^1^^,^^2^^)^. An ultrasound-guided transrectal biopsy was performed, and the histopathological study revealed abundant, benign mature smooth muscle, with scanty bistratified columnar epithelium, consistent with seminal vesicle leiomyoma (Figure 1D). In order to differentiate seminal vesicle leiomyoma from other stromal tumors, such as leiomyosarcoma and mixed epithelial stromal tumor, an immunohistochemical study was performed, which demonstrated a lack of nuclear marker (Ki-67) protein expression, as well as negativity for estrogen, progesterone, and prostate specific antigen receptors.

**Figure 1:**
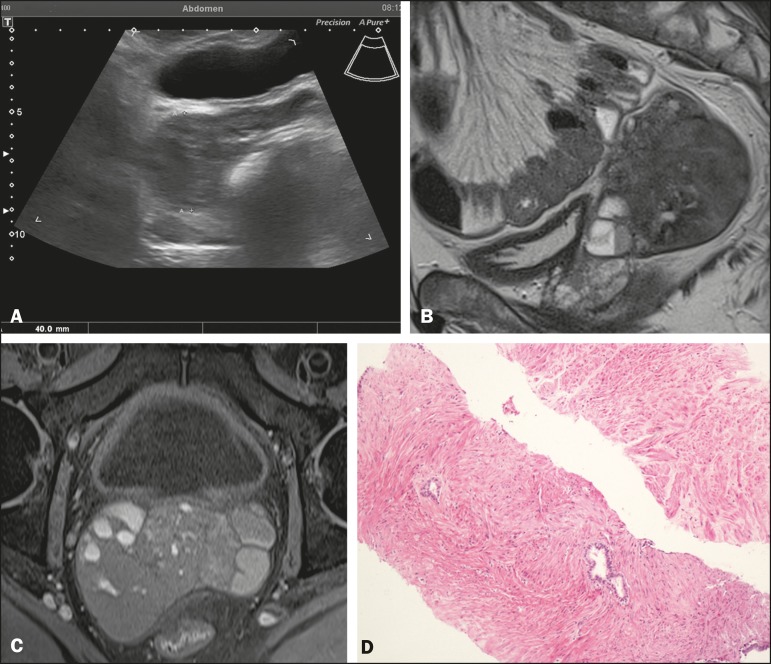
**A:** Transabdominal pelvic ultrasound showing a well-defined solid hypoechoic lesion, measuring 4.0 cm, in the right seminal vesicle space. **B:** Histological slide showing abundant, benign mature smooth muscle, with scanty bistratified columnar epithelium. **C:** Sagittal T2-weighted MRI sequence showing a well-defined, heterogeneous expansile lesion with predominantly low signal intensity. Note also the fluid-fluid level. **D:** Axial T1- weighted fast spin-echo MRI sequence showing a solid heterogeneous lesion with its epicenter in the right seminal vesicle and a predominantly isointense signal.

Genitourinary tumors have recently been reported in the radiology literature of Brazil^(^^2^^-^^7^^)^. Leiomyoma is a benign smooth muscle tumor found in the uterus and in the gastrointestinal tract. Despite the high incidence of leiomyoma in the female genital tract, it is uncommon in the male genitourinary tract, only a few cases having been reported in the literature^(^^8^^)^. Leiomyomas can proliferate in any tissue composed of smooth muscle and separated by any amount of connective tissue. According to some studies, leiomyoma originates from the Wolffian and Müllerian ducts, and the immunohistochemical differentiation remains controversial^(^^9^^)^.

Seminal vesicles are extraperitoneal glands of the male reproductive system that secrete fluid containing enzymes^(^^10^^)^. The most common changes to seminal vesicles are agenesis and cystic anomalies. Primary seminal vesicle neoplasms, which are extremely rare, include leiomyoma, cystadenoma, angioendothelioma, teratoma, schwannoma, and paraganglioma^(^^9^^,^^10^^)^. Patients with seminal vesicle leiomyoma are often asymptomatic, although lumbar pain, polyuria, dysuria, perineal pain, and infertility may occur^(^^11^^)^.

Epithelial lesions, including secondary neoplasms such as carcinoma of the prostate, bladder, rectum and primary adenosarcoma, constitute the most common presentation of seminal vesicle leiomyoma. Typical MRI findings include lesions with a hypointense signal in T2-weighted sequences, obliteration of adjacent tissues, and loss of the normal seminal vesicle architecture. The stromal lesions have a solid-cystic composition and variable signal intensity^(^^12^^)^.

Extra-adrenal paragangliomas, which are rare neuroendocrine tumors, present as well-defined lesions with a hypointense signal in T2-weighted sequences and post-contrast enhancement, some showing heterogeneity with necrotic areas. Schwannomas show a hypointense signal in T2-weighted sequences and intense post-contrast enhancement. In contrast, cystadenomas present as a well-defined, usually unilateral, multilocular cystic mass with a hypointense signal in T2-weighted sequences and discrete enhancement^(^^9^^,^^10^^,^^13^^,^^14^^)^.

Solid seminal vesicles lesions are quite rare and have poorly specific imaging characteristics. However, we can rely on the well-established diagnostic parameters of other abdominal solid lesions, such as ovarian and renal tumors, as a line of reasoning for the suspicion of benignity.

## References

[1] D'Ippolito G, Lima ACM, Peddi Neto L (2006). Neoplasias sólidas de ovário: análise
sistematizada e ensaio iconográfico. Rev Imagem.

[2] Sousa CSM, Viana IL, Miranda CLVM (2017). Hemangioma of the urinary bladder: an atypical
location. Radiol Bras.

[3] Leapman MS, Wang ZJ, Behr SC (2017). Impact of the integration of proton magnetic resonance imaging
spectroscopy to PI-RADS 2 for prediction of high grade and high stage
prostate cancer. Radiol Bras.

[4] Fernandes AM, Paim BV, Vidal APA (2017). Pheochromocytoma of the urinary bladder. Radiol Bras.

[5] Espindola APBP, Amorim VB, Koch HA (2017). Atypical presentation of mature cystic teratoma ("floating
balls"). Radiol Bras.

[6] Lima LLA, Parente RCM, Maestá I (2016). Clinical and radiological correlations in patients with
gestational trophoblastic disease. Radiol Bras.

[7] Manikkavasakar S, Ramachandram A, Ramalho M (2016). Malignant uterine disease with concurrent miometrial contraction
at MRI: a possible source of overstaging. Radiol Bras.

[8] Arnold SJ, Lin FC, Eldersveld JM (2016). Seminal vesicle leiomyoma mimicking extra-prostatic extension of
prostatic adenocarcinoma. Urol Case Rep.

[9] Shaikh AS, Bakhshi GD, Khan AS (2013). Leiomyoma of the seminal vesicle: a rare case. Clin Pract.

[10] Reddy MN, Verma S (2014). Lesions of the seminal vesicles and their MRI
characteristics. J Clin Imaging Sci.

[11] Shiotani T, Kawai N, Sato M (2009). Leiomyoma of the seminal vesicle. Jpn J Radiol.

[12] Kim B, Kawashima A, Ryu JA (2009). Imaging of the seminal vesicle and vas deferens. Radiographics.

[13] Dagur G, Warren K, Suh Y (2016). Detecting diseases of neglected seminal vesicles using imaging
modalities: a review of current literature. Int J Reprod Biomed (Yazd).

[14] Zhu JG, Chen WH, Xu SX (2013). Cystadenoma in a seminal vesicle is cured by laparoscopic
ablation. Asian J Androl.

